# Expert clinical pharmacological advice may make an antimicrobial TDM program for emerging candidates more clinically useful in tailoring therapy of critically ill patients

**DOI:** 10.1186/s13054-022-04050-9

**Published:** 2022-06-14

**Authors:** Milo Gatti, Pier Giorgio Cojutti, Michele Bartoletti, Tommaso Tonetti, Amedeo Bianchini, Stefania Ramirez, Giacinto Pizzilli, Simone Ambretti, Maddalena Giannella, Rita Mancini, Antonio Siniscalchi, Pierluigi Viale, Federico Pea

**Affiliations:** 1 Department of Medical and Surgical Sciences, Alma Mater Studiorum University of Bologna, Via Massarenti 9, 40138 Bologna, Italy; 2 Clinical Pharmacology Unit, Department for Integrated Infectious Risk Management, IRCCS Azienda Ospedaliero-Universitaria di Bologna, Bologna, Italy; 3 Infectious Diseases Unit, Department for Integrated Infectious Risk Management, IRCCS Azienda Ospedaliero-Universitaria di Bologna, Bologna, Italy; 4 Anesthesia and Intensive Care Medicine, IRCCS Azienda Ospedaliero-Universitaria di Bologna, Bologna, Italy; 5 Division of Anesthesiology, Department of Anesthesia and Intensive Care, IRCCS Azienda Ospedaliero-Universitaria di Bologna, Bologna, Italy; 6 LUM Metropolitan Laboratory, AUSL Bologna, Bologna, Italy; 7 Operative Unit of Microbiology, Department for Integrated Infectious Risk Management, IRCCS Azienda Ospedaliero-Universitaria di Bologna, Bologna, Italy

**Keywords:** Expert clinical pharmacological advice program, Critically ill patients, Personalized antimicrobial therapy, Dosing adjustments, Transplant ICU, General ICU, Turnaround time

## Abstract

**Background:**

Therapeutic drug monitoring (TDM) may represent an invaluable tool for optimizing antimicrobial therapy in septic patients, but extensive use is burdened by barriers. The aim of this study was to assess the impact of a newly established expert clinical pharmacological advice (ECPA) program in improving the clinical usefulness of an already existing TDM program for emerging candidates in tailoring antimicrobial therapy among critically ill patients.

**Methods:**

This retrospective observational study included an organizational phase (OP) and an assessment phase (AP). During the OP (January–June 2021), specific actions were organized by MD clinical pharmacologists together with bioanalytical experts, clinical engineers, and ICU clinicians. During the AP (July–December 2021), the impact of these actions in optimizing antimicrobial treatment of the critically ill patients was assessed. Four indicators of performance of the TDM-guided real-time ECPA program were identified [total TDM-guided ECPAs July–December 2021/total TDM results July–December 2020; total ECPA dosing adjustments/total delivered ECPAs both at first assessment and overall; and turnaround time (TAT) of ECPAs, defined as optimal (< 12 h), quasi-optimal (12–24 h), acceptable (24–48 h), suboptimal (> 48 h)].

**Results:**

The OP allowed to implement new organizational procedures, to create a dedicated pathway in the intranet system, to offer educational webinars on clinical pharmacology of antimicrobials, and to establish a multidisciplinary team at the morning bedside ICU meeting. In the AP, a total of 640 ECPAs were provided for optimizing 261 courses of antimicrobial therapy in 166 critically ill patients. ECPAs concerned mainly piperacillin–tazobactam (41.8%) and meropenem (24.9%), and also other antimicrobials had ≥ 10 ECPAs (ceftazidime, ciprofloxacin, fluconazole, ganciclovir, levofloxacin, and linezolid). Overall, the pre–post-increase in TDM activity was of 13.3-fold. TDM-guided dosing adjustments were recommended at first assessment in 61.7% of ECPAs (10.7% increases and 51.0% decreases), and overall in 45.0% of ECPAs (10.0% increases and 35.0% decreases). The overall median TAT was optimal (7.7 h) and that of each single agent was always optimal or quasi-optimal.

**Conclusions:**

Multidisciplinary approach and timely expert interpretation of TDM results by MD Clinical Pharmacologists could represent cornerstones in improving the cost-effectiveness of an antimicrobial TDM program for emerging TDM candidates.

## Background

Sepsis is a common occurrence among patients admitted to the intensive care unit (ICU) and may account for high mortality rate and massive antibiotic consumption [1–3]. Up to 70% of critically ill patients may receive at least one antimicrobial treatment during ICU stay [3]. Antimicrobial treatment in the critically ill patients may be challenged by the emergence of multidrug-resistant (MDR) pathogens and by the complex pathophysiology of sepsis, which may alter the pharmacokinetic behavior of hydrophilic drugs [4–6]. Early appropriate antimicrobial treatment was shown to decrease the mortality rate among septic patients [7, 8]. To be appropriate, antimicrobial exposure should maximize microbial killing, minimize development of resistance, and prevent drug overexposure-related adverse events [4, 9, 10].

Indeed, the Surviving Sepsis Campaign guidelines recommended that antimicrobial dosing strategies should be optimized in septic critically ill patients on the basis of well-recognized and drug-specific pharmacokinetic/pharmacodynamic (PK/PD) principles [11]. In this scenario, real-time optimization of antibiotic exposure may play a key role. Therapeutic drug monitoring (TDM) may represent an invaluable tool in making intensive care physicians sure that optimal antimicrobial PK/PD targets have been promptly achieved in each single patient and then maintained throughout the whole treatment period. In a recent position paper, an expert panel of international researchers agreed that TDM is the only safe and effective way for optimizing properly treatment with several antimicrobials in the critically ill patients, namely with beta-lactams, glycopeptides, aminoglycosides, linezolid, and/or voriconazole [12]. Likewise, guidelines from the French Society of Pharmacology and Therapeutics strongly suggested that beta-lactams should be administered by continuous infusion in septic critically ill patients and that exposure should be personalized by means of an adaptive TDM strategy [13]. Adaptive TDM is an approach that allows to adjust the dosage of a given drug on the basis of expert interpretation of the TDM results by considering the site of infection, the patient’s underlying conditions, and/or eventual iatrogenic interventions. This approach should be considered especially relevant whenever major fluctuations of renal function may affect stable and appropriate drug exposure [14–16].

The use of adaptive TDM is a quite well-consolidated approach for aminoglycosides and glycopeptides, but it has been argued that for other antimicrobials, like for example beta-lactams and azoles, extensive application is still burdened by many barriers. Availability of TDM equipment set up by bioanalytical experts with analytical methods having short turnaround times and appropriate interpretation of TDM results by clinical pharmacological experts for prompt dosing adaptation could be considered the two most relevant ones [6, 17–19].

The aim of this study was to assess the impact of a newly established expert clinical pharmacological advice (ECPA) program in improving the clinical usefulness of an already existing TDM program for emerging candidates in tailoring antimicrobial therapy among critically ill patients.

## Methods

This retrospective observational study was carried out between January 2021 and December 2021 at the IRCCS Azienda Ospedaliero-Universitaria of Bologna, which is a 1362-bed tertiary care university hospital where a Clinical Pharmacology Unit (CPU) was newly established in January 2021. The CPU was included in the Department for Integrated Infectious Risk Management and has been provided with three MD clinical pharmacologists.

The study period was divided into two subsequent phases, namely the organizational phase and the assessment phase. The organizational phase corresponded to the first 6 months of activity (January–June 2021). During this period, the major commitments of the MD clinical pharmacologists were focused on how to take actions for making the already existing TDM program of antimicrobials more clinically useful in tailoring antimicrobial therapy among critically ill patients. A state of the art of the TDM program was carried out and specific actions were organized by involving bioanalytical experts, clinical engineers, and ICU clinicians. In regard to the TDM program, bioanalytical experts were located in the Unique Metropolitan Laboratory [LUM] and their role was to set methods for measuring drug concentrations by means of mass spectrometry; clinical engineers had an important role in organizing the logistic and the optimal pathways in the intranet hospital system.

The assessment phase corresponded to the subsequent 6 months (July–December 2021). During this period, the impact of the actions taken during the organizational phase was assessed by analyzing the overall activity and the performance of the ECPA program. All the critically ill patients admitted in this timeframe in the 13-bed general ICU and in the 8-bed post-transplant ICU were retrospectively retrieved. Only patients with at least one TDM-guided real-time ECPA for optimizing antimicrobial treatment during ICU stay were included. For each patient, the following demographic and clinical features were collected: age, gender, weight, height, body mass index (BMI), ICU admission diagnosis, measured or estimated creatinine clearance (CLCr), need for mechanical ventilation, PaO_2_/FiO_2_ ratio, need and dosing of vasopressors, need and effluent flow rate of CRRT, Sequential Organ Failure Assessment (SOFA) score [20], occurrence of augmented renal clearance (ARC) [21], site of infection, bacterial clinical isolate and antimicrobial susceptibility (if available), antimicrobial treatment and dosing, date and time of TDM assessment, dosing adjustments recommended in ECPAs, and ICU mortality rate.

Four indicators of performance were identified for assessing the impact of the ECPA program. First, the ratio between the total number of TDM-guided ECPAs provided in this period and the TDM results provided in the 6-month period immediately preceding CPU establishment (namely July–December 2020) was assumed as indicator of the overall clinical impact of the ECPA program. Second, the ratio at first TDM instance between the total number of ECPA recommending dosing adjustments and the total number of delivered ECPAs was assumed as indicator of performance of the usefulness of the ECPA program in allowing early optimization of antimicrobial exposure. Third, the overall ratio between the total number of ECPA recommending dosing adjustments and the total number of delivered ECPAs was assumed as indicator of performance of the overall usefulness of the ECPA program in allowing optimization of antimicrobial exposure during the whole treatment period. Fourth, the turnaround time (TAT) of the final antimicrobial ECPA (defined as the timeframe elapsed between the delivery of TDM blood sample to the LUM and the publication of the final TDM-guided ECPA in the hospital intranet system) was assumed as indicator of performance of timely usefulness of the ECPA program in allowing prompt dosing adaptation. The TAT was defined as optimal when < 12 h, quasi-optimal when between 12 and 24 h, acceptable when between 24 and 48 h, and suboptimal when > 48 h.

Data were expressed as mean ± standard deviation or median and interquartile range (IQR) according to data distribution, whereas categorical variables were expressed as count and percentage.

## Results

### Organizational phase

The state of the art showed that the already existing TDM program of antimicrobials for emerging candidates of the LUM of Bologna was firstly implemented by the bioanalytical experts in 2014. In January 2021, this program allowed the measurement of serum concentrations of 15 different drugs [9 antibiotics (ampicillin, ampicillin–sulbactam, ceftazidime, cefepime, meropenem, piperacillin–tazobactam, linezolid, levofloxacin, ciprofloxacin), 4 antifungals (fluconazole, voriconazole, posaconazole, and isavuconazole), and 2 antivirals (ganciclovir, acyclovir)] by means of validated liquid chromatography-tandem mass spectrometry (LC–MS/MS) methods, which requested fully manual operator-dependent procedures. Kits of reagents and analytical columns were provided by commercial companies [22, 23]. At that time, it was verified that TDM sessions were carried out twice- or thrice–weekly with a TAT of approximately 48–72 h, and that TDM results were delivered to the applicant clinician simply as drug concentrations without any expert interpretation and/or suggestion on how to perform dosing adaptation.

The second step was to take actions together with bioanalytical experts, clinical engineers, and ICU clinicians in order to define the optimal pathways for providing a cost-effective TDM-guided ECPA program for optimizing antimicrobial treatment via the hospital intranet system.

In regard to bioanalytical experts, new organizational procedures were implemented for improving the reliability of TDM blood sampling, the frequency of TDM sessions and the TAT of the TDM-guided ECPA. It was agreed that TDM sessions would have run daily Monday to Friday, that blood sampling for first TDM assessment should have been collected after 24–48 h from starting treatment, and that all samples delivered at the LUM by 11:00 a.m. should have been processed within the afternoon of the same day. Otherwise, they would have been processed the day after.

In regard to clinical engineers, they created a dedicated pathway in the intranet system to speed up the process as much as possible. This allowed TDM results to be readily available to the MD Clinical Pharmacologists who provided promptly ICU clinicians with a timely TDM-guided ECPA for dosing adaptation.

The structure of the TDM-guided ECPA form was organized as an expert interpretation of the TDM result based on some specific underlying conditions. Dosing adaptation was defined by taking into account the in vitro susceptibility of the suspected or the documented bacterial pathogens, the site of infection, the pathophysiological characteristics of each single patient [e.g., body mass index, measured or estimated CLCr, the presence of sepsis or septic shock and/or of other co-morbidities, the eventual application of renal replacement therapy], and/or the potential of drug–drug interactions due to co-treatments [24]. In regard to the demographic and clinical features of the patient, a pre-defined mask was set in the intranet system. In this way, the ICU clinician having in charge a given patient filled it with the fundamental info at time of ECPA application (i.e., weight, height, site of infection, date of starting antimicrobial therapy, posology and time of last administration, time of blood sample collection, underlying diseases, co-treatments). In regard to the in vitro susceptibility of the bacterial pathogens, the PK/PD targeting of each antimicrobial was set depending on whether empirical or targeted therapy was the case. As referral MIC value, it was considered the EUCAST clinical breakpoint of the suspected pathogens in case of empirical treatment, and the actual MIC value of the clinical isolate in case of targeted therapy. This approach was thought useful at maximizing as much as possible the probability of achieving optimal PK/PD target of antimicrobials in all of the clinical scenarios. Each ECPA usually took 10–30 min in relation to case-mix complexity. The desired PK/PD targets of the different antimicrobials are summarized in Table 1. They were set for maximizing clinical efficacy, and for minimizing either the risk of resistance development [12] or that of toxicity [25–31].

**Table 1:** Desired PK/PD target, thresholds for toxicity, and TDM-guided dosage adjustments of antimicrobials included in the expert clinical pharmacological advice (ECPA) program

Antimicrobial	Desired target	Threshold for toxicity	Dosage adjustment
Piperacillin–tazobactam	*C*_ss_ 4–8 × MIC (for piperacillin)	*C*_min_ > 361 mg/L (neurotoxicity) [25]	*Decrease* 50% if *C*_ss_ > 10 × MIC 25% if *C*_ss_ 8–10 × MIC *Increase* 50% if *C*_ss_ < 2 × MIC 25% if *C*_ss_ 2–4 × MIC
Meropenem	*C*_ss_ 4–8 × MIC	*C*_min_ > 64.2 mg/L (neurotoxicity) [25]	*Decrease* 50% if *C*_ss_ > 10 × MIC 25% if *C*_ss_ 8–10 × MIC *Increase* 50% if *C*_ss_ < 2 × MIC 25% if *C*_ss_ 2–4 × MIC
Ceftazidime	*C*_ss_ 4–8 × MIC	NA	*Decrease* 50% if *C*_ss_ > 10 × MIC 25% if *C*_ss_ 8–10 × MIC *Increase* 50% if *C*_ss_ < 2 × MIC 25% if *C*_ss_ 2–4 × MIC
Ampicillin Ampicillin–Sulbactam	*C*_ss_ 4–8 × MIC (for ampicillin)	NA	*Decrease* 50% if *C*_ss_ > 10 × MIC 25% if *C*_ss_ 8–10 × MIC *Increase* 50% if *C*_ss_ < 2 × MIC 25% if *C*_ss_ 2–4 × MIC
Cefepime	*C*_ss_ 4–8 × MIC	*C*_min_ > 36 mg/L (neurotoxicity) [26]	*Decrease* 50% if *C*_ss_ > 10 × MIC 25% if *C*_ss_ 8–10 × MIC *Increase* 50% if *C*_ss_ < 2 × MIC 25% if *C*_ss_ 2–4 × MIC
Linezolid	*C*_min_ 2–8 mg/L	*C*_min_ > 8 mg/L (thrombocytopenia) [29, 30]	*Decrease* 50% if *C*_min_ > 15 mg/L 25% if *C*_min_ 8–15 mg/L *Increase* 50% if *C*_min_ < 1 mg/L 25% if *C*_min_ 1–2 mg/L
Levofloxacin	*C*_max_ 10 × MIC *C*_min_ < 3 mg/L	NA	*Decrease* every 36–48 h if *C*_min_ > 2 mg/L *Increase* 25% if *C*_max_ < 10 × MIC
Ciprofloxacin	*C*_max_ 10 × MIC *C*_min_ < 2 mg/L	NA	*Decrease* 25% if *C*_min_ > 2 mg/L *Increase* 25% if *C*_max_ < 10 × MIC
Fluconazole	*C*_min_ 10–20 mg/L	NA	*Decrease* 50% if *C*_min_ > 50 mg/L 25% if *C*_min_ 30–50 mg/L *Increase* 25% if *C*_min_ < 10 mg/L
Voriconazole	*C*_min_ 1–3 mg/L	*C*_min_ > 3–4 mg/L (hepatotoxicity) [28]	*Decrease* stop if *C*_min_ > 8–10 mg/L 25–50% if *C*_min_ 3.5–8 mg/L *Increase* every 6–8 h if *C*_min_ < 1 mg/L
Posaconazole	*C*_min_ 1–3 mg/L	*C*_min_ > 3 mg/L (pseudohyperaldosteronism) [31]	*Decrease* 25–50% if *C*_min_ > 3 mg/L *Increase* every 12 h if *C*_min_ < 1 mg/L
Isavuconazole	*C*_min_ 1–7 mg/L	*C*_min_ > 5.1 mg/L (gastrointestinal disorders) [27]	*Decrease* 25–50% if *C*_min_ > 5 mg/L *Increase* 25–50% if *C*_min_ < 1 mg/L
Ganciclovir/Valganciclovir	*C*_min_ 0.7–2 mg/L	NA	*Decrease* stop if *C*_min_ > 5 mg/L 25–50% if *C*_min_ 2–5 mg/L *Increase* every 6–8 h if *C*_min_ < 0.5 mg/L
Acyclovir	*C*_min_ 1–3 mg/L	NA	*Decrease* stop if *C*_min_ > 5 mg/L 25–50% if *C*_min_ 3–5 mg/L *Increase* every 6 h if *C*_min_ < 0.5 mg/L

*AUC* area under concentration–time curve, *C*_*max*_ peak concentration, *C*_*min*_ trough concentration, *C*_*ss*_ steady-state concentration, *CI* continuous infusion, *ECPA* expert clinical pharmacology advice, *MIC* minimum inhibitory concentration, *NA* not available, *TDM* therapeutic drug monitoring

**Fig. 1:**
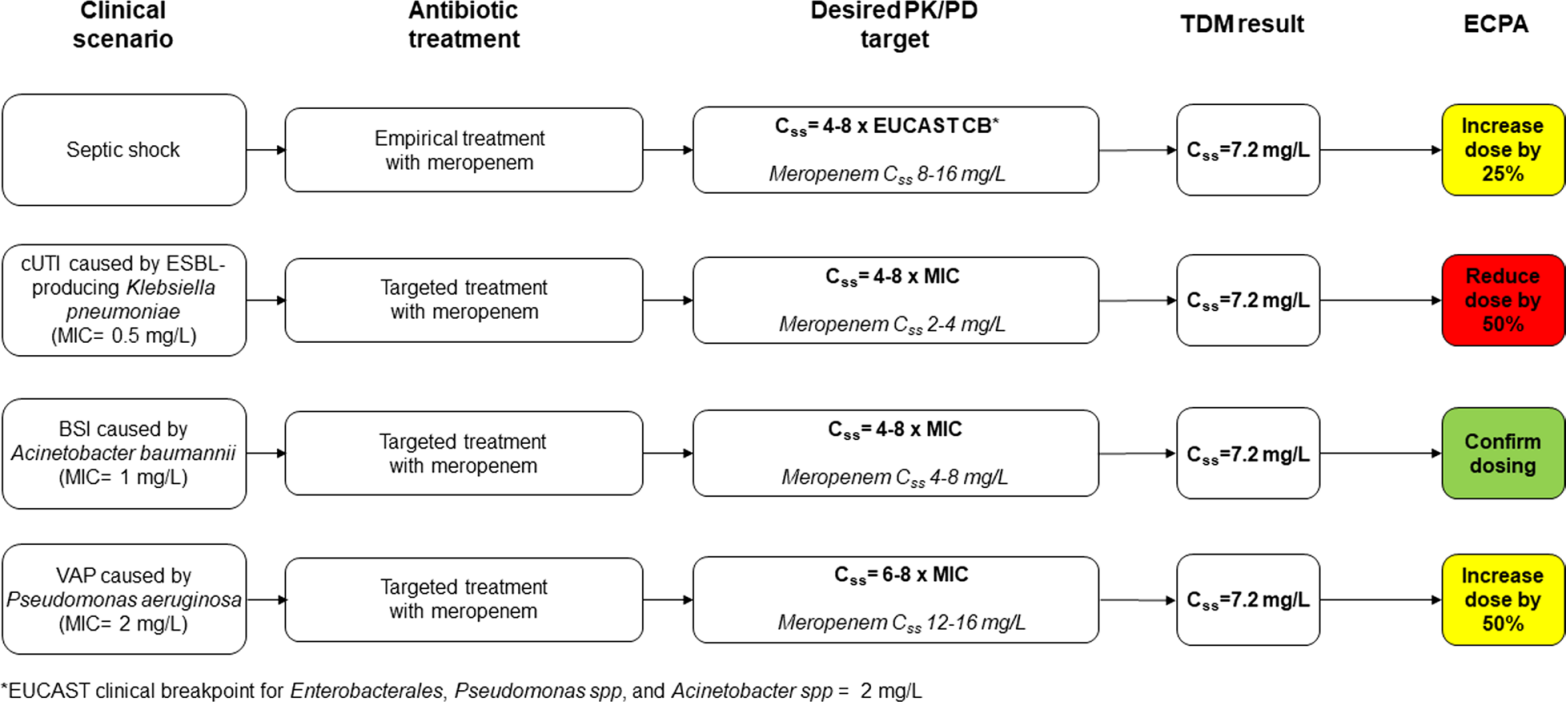
Algorithms for optimizing meropenem dosing schedule according to different scenarios of empirical or targeted therapy. Dosing adjustments are implemented according to Table 1

**Fig. 2:**
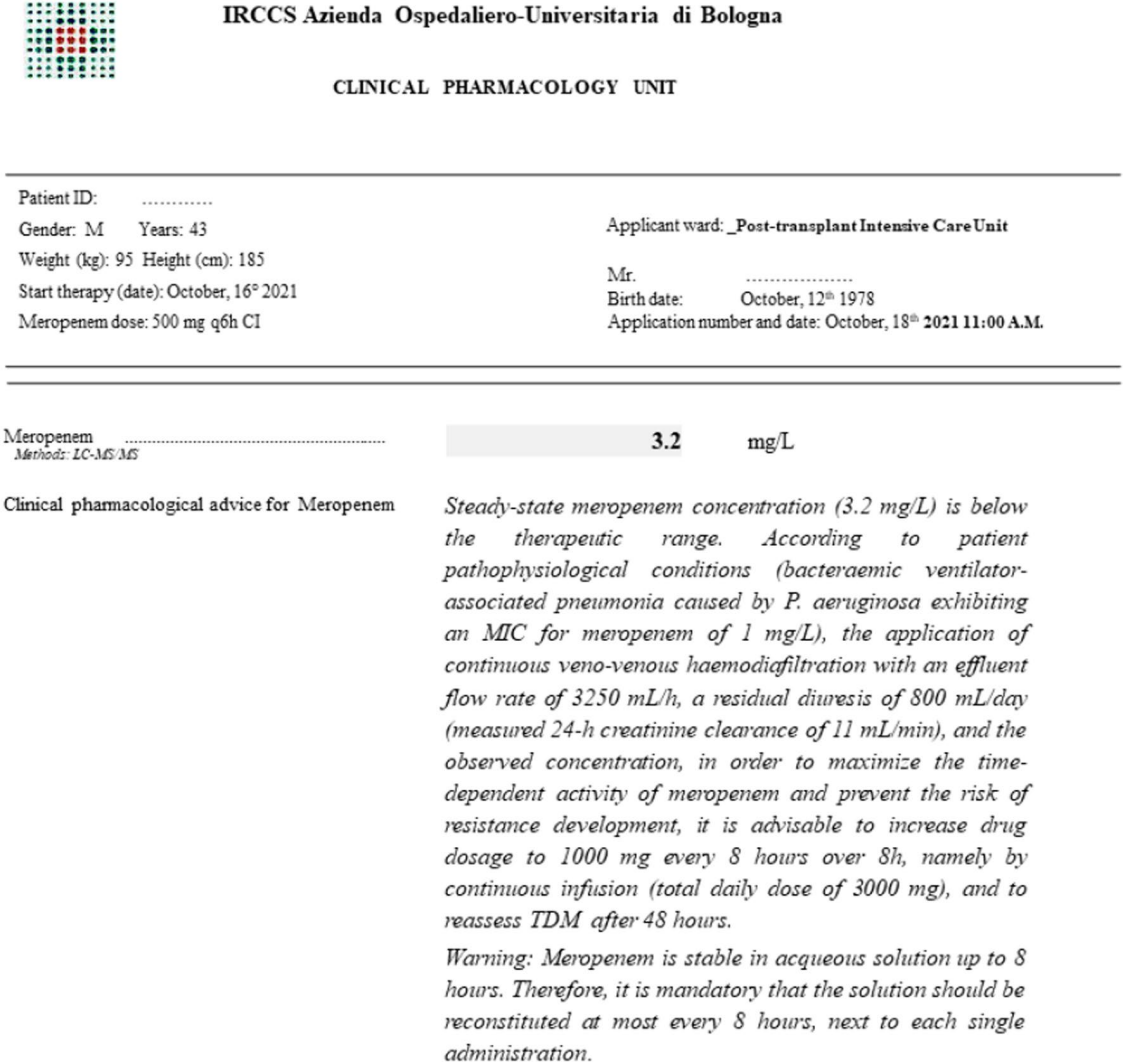
Example of a TDM-guided expert clinical pharmacological advice for personalizing antibiotic treatment with meropenem in a critically ill patient

Different scenarios of dosing adaptation during empirical and targeted therapy with meropenem are depicted in Fig. 1 [9, 32]. An example of TDM-guided ECPA is shown in Fig. 2.

In this latter regard, it is worth noting that in case of ventilator-associated pneumonia (VAP) a more aggressive strategy is pursued compared to bloodstream and/or urinary tract infections in order to address the issue of the limited penetration rate of beta-lactams into the epithelial lining fluid. In VAP, the PK/PD target is usually narrowed to the upper part of the range (*C*_ss_/MIC ratio of 6–8 instead of 4–8) in order to maximize as much as possible that antimicrobial efficacy at the infection site.

In regard to ICU clinicians, two complementary activities were carried out with the intent of increasing the awareness that a well-structured ECPA program could have had in optimizing TDM-guided antimicrobial exposure in the critically ill patients. First, some educational webinars on clinical pharmacology of antimicrobials in the critically ill patients were provided. They were focused mainly on highlighting which pathophysiological changes occurring in this patient population may cause unpredictability of antimicrobial exposure and suboptimal attainment of PK/PD targets. Second, Monday-to-Friday attendance of the MD Clinical Pharmacologist together with the Infectious Disease consultant at the morning bedside ICU meeting was agreed. The multidisciplinary approach was established for allowing daily discussion about some major points that could have made the TDM-guided ECPA program really reliable, i.e., which critically ill patients could benefit more from the ECPA program, which timing is the best for blood sampling in relation to drug administration, how to take care of clinical/laboratory evolution and of pathophysiological/iatrogenic variations in each single critically ill septic patient, the need of updating microbiological culture results for targeting PK/PD of antimicrobials timely.

**Fig. 3:**
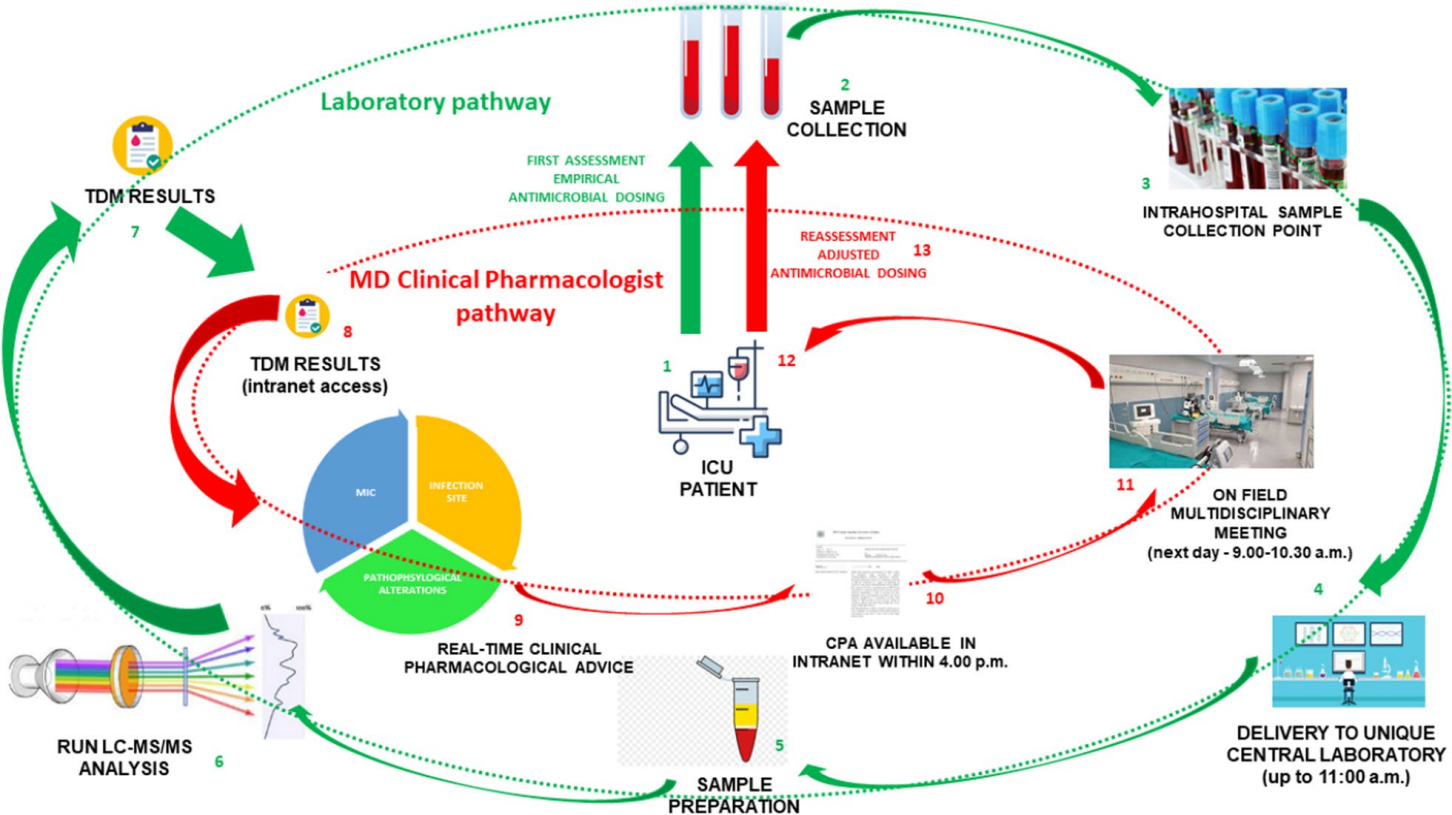
Final structured plan of the organizational phase of the TDM-based ECPA program. Two complementary pathways were identified: the laboratory pathway (in green, points 1–7), the MD clinical pharmacologist pathway (in red, points 8–14)

The final structured plan of the organizational phase of the TDM-based ECPA program is summarized in Fig. 3.

### Assessment phase

**Fig. 4:**
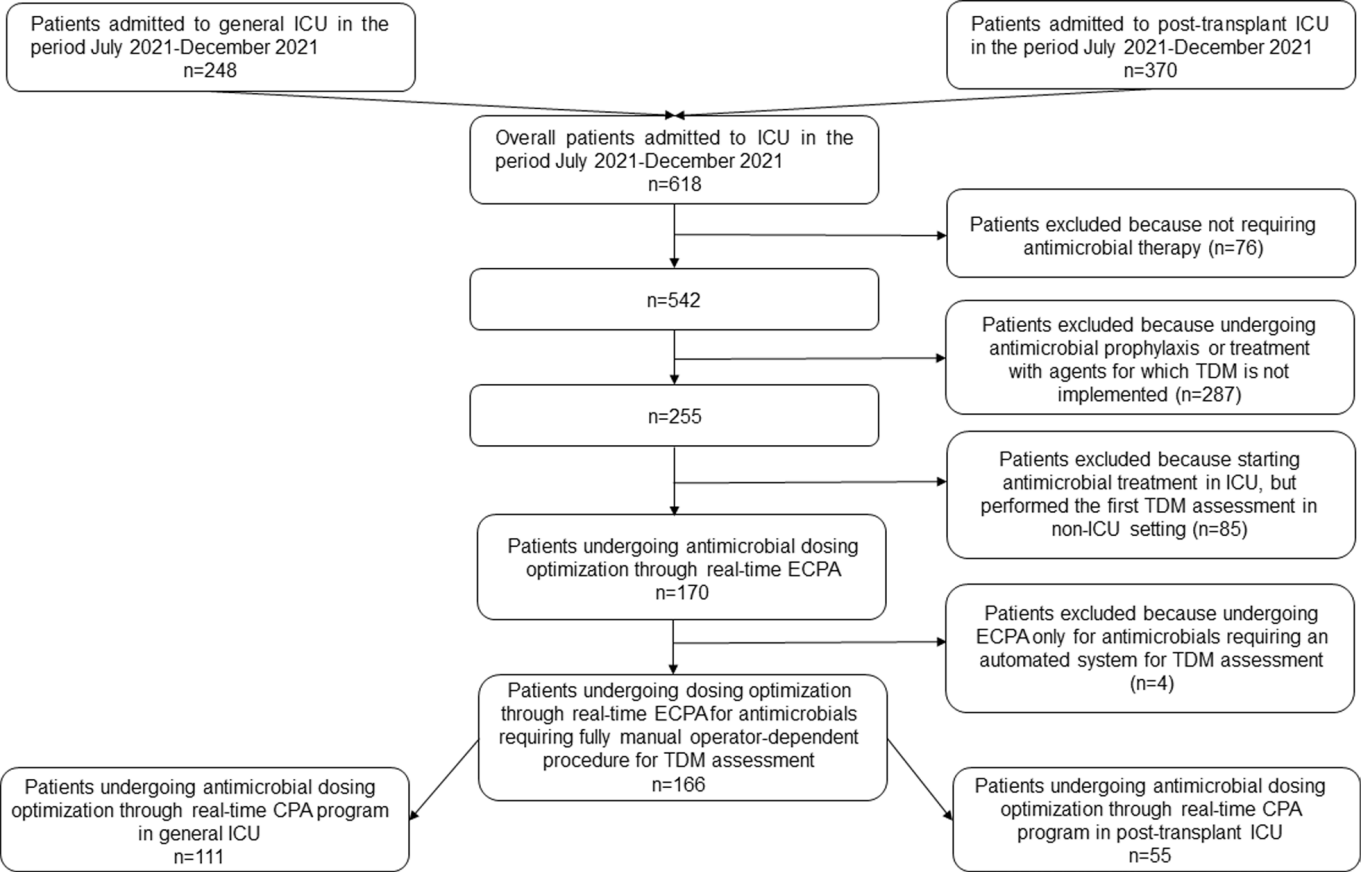
Flowchart of patient inclusion and exclusion criteria

Overall*,* during the study period 618 ICU patients were admitted in the two ICUs (248 in the general ICU and 370 in the post-transplant ICU), and 166 out of them were included in the assessment phase (111 and 55 in general and post-transplant ICU, respectively; Fig. 4).

Demographics and clinical characteristics of the included patients are reported in Table 2.

**Table 2:** Demographics and clinical characteristics of critically ill patients undergoing antimicrobial dosing optimization through real-time ECPA-guided program

*Patient demographic*	
Age (years) [median (IQR)]	66.5 (56.0–75.0)
Gender (male/female) [*n* (%)]	113/53 (68.1%/31.9%)
Body weight (kg) [median (IQR)]	75.0 (62.0–85.0)
Body mass index (kg/m^2^) [median (IQR)]	25.7 (22.0–28.3)
CL_CR_ (mL/min/1.73 m^2^)^a^ [median (IQR)]	55.0 (28.3–98)
Augmented renal clearance [*n* (%)]	16 (9.6%)
Vasopressors requirement [*n* (%)]	97 (58.4%)
Mechanical ventilation [*n* (%)]	137 (82.5%)
CRRT [*n* (%)]	44 (26.5%)
SOFA score^a^ [median (IQR)]	8 (4–12)
*Setting* [*n* (%)]	
General ICU	111 (66.9%)
Post-transplant ICU	55 (33.1%)
*Underlying disease for ICU admission* [*n* (%)]	
Acute respiratory failure	40 (24.1%)
Post-operatory sepsis	34 (20.5%)
Septic shock	29 (17.5%)
Abdominal perforation	27 (16.3%)
Solid organ transplant	11 (6.6%)
Hemorrhagic shock	6 (3.6%)
Cardiac arrest	5 (3.0%)
Other	14 (8.4%)
*Antimicrobial treatment*^b^ [*n* (%)]	
Empirical	131 (50.2%)
Targeted	128 (49.0%)
Prophylaxis	2 (0.8%)
*Antimicrobial used*^b^ [*n* (%)]	
Piperacillin–Tazobactam	109 (41.8%)
Meropenem	65 (24.9%)
Fluconazole	23 (8.8%)
Linezolid	20 (7.7%)
Levofloxacin	18 (6.9%)
Ganciclovir	8 (3.1%)
Ceftazidime	8 (3.1%)
Ciprofloxacin	5 (1.9%)
Voriconazole	3 (1.1%)
Acyclovir	2 (0.7%)
*Clinical outcome* [*n* (%)]	
ICU mortality rate	33 (19.9%)

^a^At baseline

^b^Overall, 261 different antimicrobial treatments were implemented in included patients

Data are presented as median (IQR) for continuous variables and as *n* (%) for dichotomous variables

The median age was 66.5 years (IQR 56–75 years), and male gender was prevalent (68.1%). During ICU stay, 58.4% of patients needed vasopressors, 82.5% underwent mechanical ventilation, 26.5% underwent CRRT, and 9.6% had ARC. Median SOFA score at ICU admission was 8 (IQR 4–12). Acute respiratory failure and post-surgical sepsis accounted for approximatively half ICU admission diagnosis, and mortality rate was 19.9%.

Piperacillin–tazobactam (41.8%) and meropenem (24.9%) were the two most frequent antimicrobial treatment optimized by means of the ECPA program, with similar proportions between empirical and targeted therapy. Sites of infection and bacterial isolates concerning the 128 targeted therapies that were included in the ECPA program are summarized in Table 3.

**Table 3:** Site of infections and isolated pathogens in the 128 targeted antimicrobial therapies underwent ECPA program

*Site of infections*^a^	
Pneumonia	50 (38.2%)
Bloodstream infection	34 (26.0%)
Complicated intra-abdominal infection	29 (22.1%)
Complicated urinary tract infection	9 (6.9%)
Bone and joint infection	4 (3.1%)
Necrotizing soft tissue infection	2 (1.5%)
Meningitis	2 (1.5%)
Catheter-related bloodstream infection	1 (0.7%)
*Isolated pathogens*^b^	
Gram-positive (9)	
*Staphylococcus aureus*	7 (5.1%)
*Enterococcus faecium*	2 (1.4%)
Gram-negative (115)	
*Pseudomonas aeruginosa*	31 (22.6%)
*Escherichia coli*	24 (17.5%)
*Klebsiella pneumoniae*	22 (16.1%)
*Proteus mirabilis*	7 (5.1%)
*Enterobacter cloacae complex*	7 (5.1%)
*Klebsiella aerogenes*	6 (4.3%)
*Serratia marcescens*	4 (2.9%)
*Klebsiella oxytoca*	2 (1.4%)
*Klebsiella variicola*	2 (1.4%)
*Morganella morganii*	2 (1.4%)
*Acinetobacter baumannii*	2 (1.4%)
*Proteus vulgaris*	1 (0.7%)
*Citrobacter koseri*	1 (0.7%)
*Hafnia alvei*	1 (0.7%)
*Pantoea spp*	1 (0.7%)
*Acinetobacter pittii*	1 (0.7%)
*Stenotrophomonas maltophilia*	1 (0.7%)
Anaerobes (1)	
*Bacteroides faecis*	1 (0.7%)
Atypical (4)	
*Legionella pneumophila*	4 (2.9%)
Fungi (8)	
*Candida albicans*	5 (3.7%)
*Candida glabrata*	1 (0.7%)
*Aspergillus fumigatus*	1 (0.7%)
*Aspergillus terreus*	1 (0.7%)
Virus	
CMV reactivation	1 (0.7%)

^a^Overall, 131 different site of infections were identified for the 128 targeted antimicrobial therapies

^b^Overall, 137 different pathogens were identified for the 128 targeted antimicrobial therapies

*CMV* cytomegalovirus

Data are presented as *n* (%)

Pneumonia (38.2%), bloodstream infections (26.0%), and complicated intra-abdominal infections (22.1%) accounted for almost 90% of infections. Overall, 137 bacterial pathogens were yielded. Gram-negative accounted for more than 80% of isolates. *Pseudomonas aeruginosa* was the most prevalent (22.6%), followed by *Escherichia coli* (17.5%), and *Klebsiella pneumoniae* (16.1%). Among *Enterobacterales*, the prevalence of extended-spectrum beta-lactamases (ESBLs)-producing strains was of 32.1%.

Overall, the total number of TDM-guided ECPAs delivered in the period July–December 2021 was of 640, whereas that of TDM results provided by the LUM in the period July–December 2020 was of 48. (15, 13, 11, and 9 TDM results were delivered for piperacillin–tazobactam, voriconazole, meropenem, and linezolid, respectively.) Consequently, the overall clinical impact of the ECPA program was of increasing the TDM activity by 13.3-fold during the study period.

Overall, 261 courses of antimicrobial therapy were optimized (2.5 ± 1.8 ECPAs per antimicrobial course). Piperacillin–tazobactam and meropenem accounted for 35.2% and 34.8% of the TDM-guided ECPAs, respectively. Other antimicrobials with a total number of ECPAs delivered during the study period ≥ 10 were levofloxacin, linezolid, ceftazidime, ciprofloxacin, fluconazole, and ganciclovir. Conversely, no TDM-guided ECPA was requested for ampicillin, ampicillin–sulbactam, cefepime, posaconazole, and isavuconazole.

**Fig. 5:**
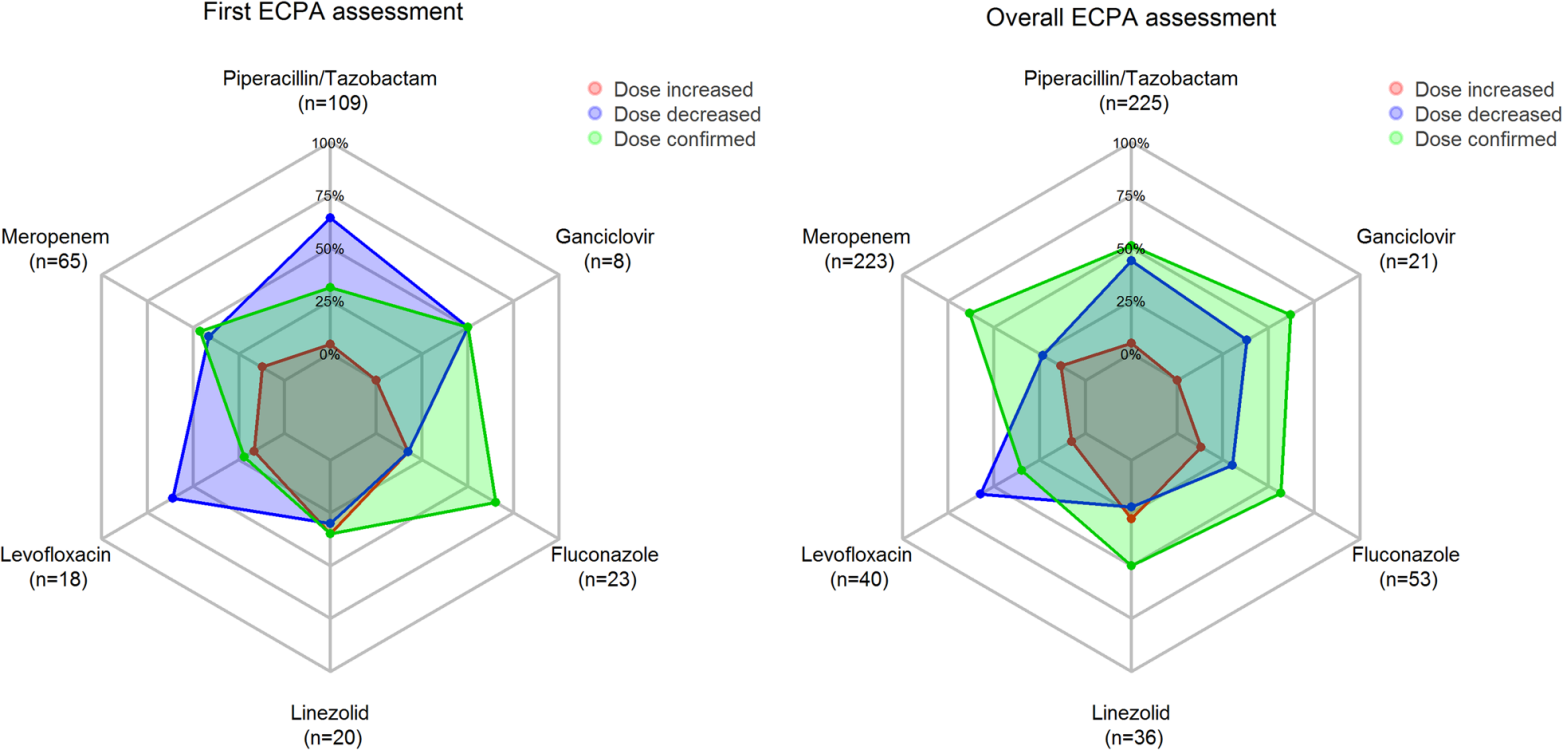
**a** Radar plot of the proportion of dosing recommendations at first TDM assessment for those antimicrobials with a total number of delivered ECPAs ≥ 20 during the study period. **b** Radar plot of the overall proportion of dosing recommendations for those antimicrobials with a total number of delivered ECPAs ≥ 20 during the study period

At first TDM assessment (Fig. 5a), the ECPA program recommended dosing decreases in 51.0% of cases and increases in other 10.7%. This is helpful in allowing early optimization of antimicrobial exposure in 61.7% (161/261) of implemented treatments. Dosage increases concerned mainly linezolid (35.0%) and fluconazole (17.4%), whereas decreases concerned mainly piperacillin–tazobactam (64.2%) and levofloxacin (61.1%).

Overall, during the whole treatment period (Fig. 5b) the ECPA program was helpful in recommending dosing adaptation of antimicrobial exposure in 45.0% of cases (288/640), being 35.0% decreases and 10.0% increases. Dosage increases concerned mainly linezolid (27.8%) and meropenem (13.5%), whereas dose reductions were needed mainly for levofloxacin (57.5%), ciprofloxacin (50.0%), and piperacillin–tazobactam (44.0%). The need of dosing adaptation was more frequent among patients receiving empirical treatment than in those having targeted therapy (48.6% vs. 41.8%).

**Fig. 6:**
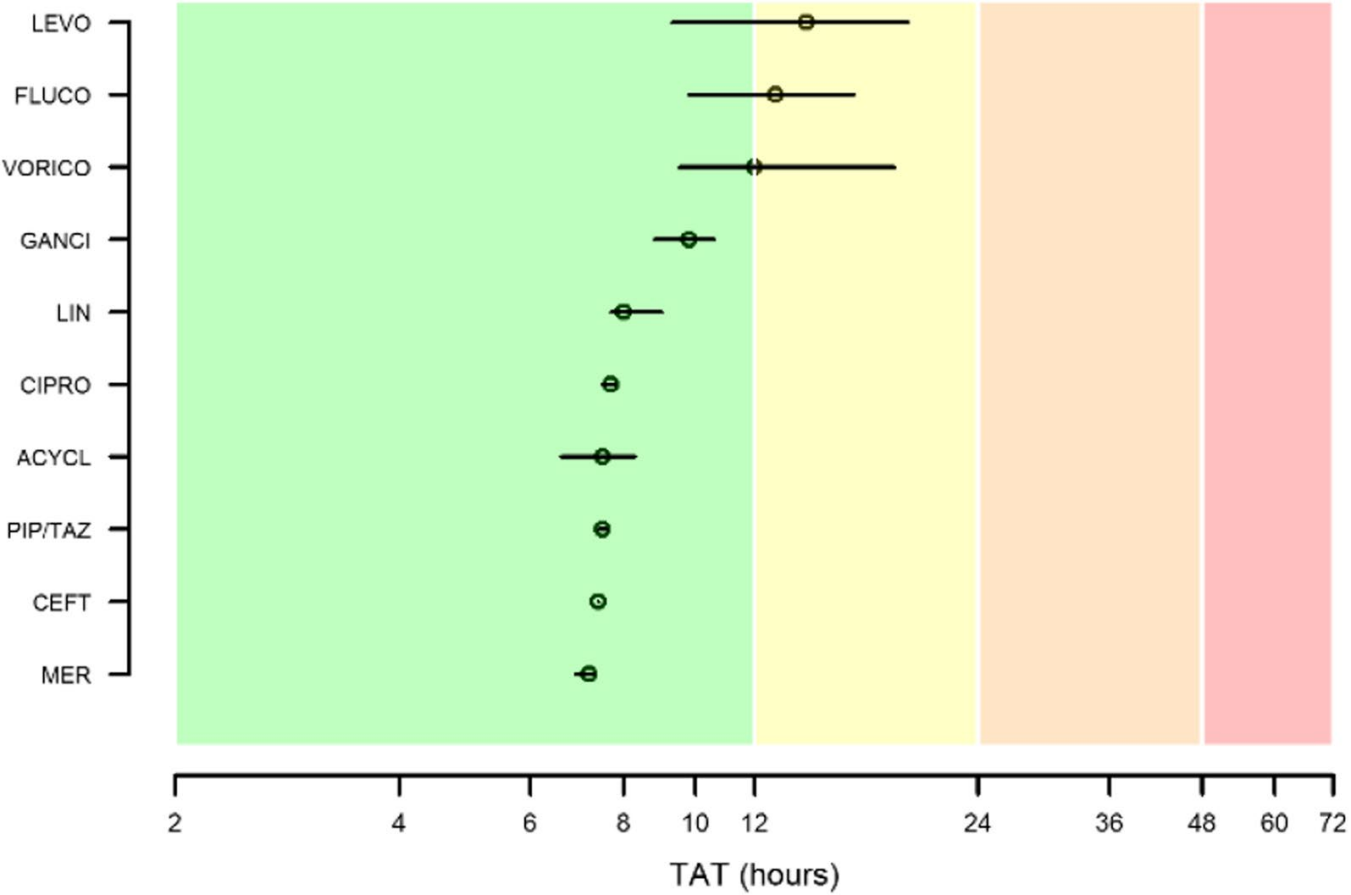
Median and interquartile range of the turnaround times (TATs) of the TDM-guided ECPAs for the included antimicrobials

The overall median TAT of the TDM-guided ECPAs was 7.7 h (IQR 7.6–9.3 h). In regard to each single agent (Fig. 6), median TAT was optimal for meropenem (7.2 h; IQR 6.9–7.3 h), ceftazidime (7.4 h; IQR 7.4–7.4 h), piperacillin–tazobactam (7.5 h; IQR 7.4–7.6 h), acyclovir (7.5 h; IQR 6.6–8.3 h), ciprofloxacin (7.7 h; 7.5–7.8 h), linezolid (8.0 h; IQR 7.7–9.0 h), and ganciclovir (9.8 h; IQR 8.8–10.6 h), and quasi-optimal for voriconazole (12.0 h; IQR 9.5–18.5 h), fluconazole (12.8 h; IQR 9.8–16.3 h), and levofloxacin (14.1 h; IQR 9.3–19.3 h). No antimicrobial had acceptable or suboptimal median TAT (namely > 24 h).

## Discussion

To the best of our knowledge, this is the first study that described how to implement the organization of a TDM-based ECPA program of emerging TDM candidates, and that assessed the program usefulness in tailoring therapy in a large cohort of critically ill patients admitted in the ICUs of a tertiary university hospital.

Several guidelines currently recommend that antimicrobial dosing in critically septic patients should be optimized according to well-recognized PK/PD principles and guided by adaptive TDM strategy [11–13]. Likewise, several evidences supported the role of TDM as an invaluable tool for maximizing the achievement of optimal PK/PD targets of antimicrobials in the ICU setting [14–16, 33]. However, no study explored how to organize a TDM-guided ECPA program of antimicrobials focused on delivering timely dosing adaptation in ICU patients. Interestingly, our findings suggest that the idea of the MD Clinical Pharmacologists of involving bioanalytical experts, clinical engineers, and ICU clinicians in the brainstorming was successful in taking proper actions for finalizing a well-structured program.

The achievement of this goal was made possible thanks to the close cooperation and integration between the expertise of the bioanalytical experts and that of the MD Clinical Pharmacologists. The organizational efforts of the bioanalytical experts made possible having Monday-to-Friday TDM sessions for 15 different antimicrobials with very short TATs of TDM results. The expert and comprehensive clinical interpretation of the TDM results by the MD clinical pharmacologists was helpful in increasing the awareness of the intensivists about the importance of the TDM-based ECPA program and in making feasible, thanks to the intranet system, timely dosing adaptation for optimizing antimicrobial exposure in the critically ill patients. Daily attendance at the ICU bedside morning multidisciplinary briefing reinforced the direct interaction between the MD clinical pharmacologists and the intensive care physicians in the context of a dedicated multidisciplinary taskforce and concurred in making the ECPA program more clinically helpful in the ICU setting.

The innovative feature of this approach may represent a paradigm shift in the TDM era. Noteworthy, the TDM-based ECPA program was organized in two separate but complementary pathways, namely the laboratory pathway and the clinical pharmacology pathway. In this regard, a recent survey conducted in 82 Australian hospitals reported that TDM of antimicrobials for emerging TDM candidates (namely beta-lactam antibiotics, anti-tuberculous agents, flucytosine, fluoroquinolones, ganciclovir, HIV drugs, linezolid and teicoplanin) was available only in 25% of them [17]. Of note, TDM of beta-lactams was available only in 6% of hospitals, the TAT exceeded 24 h in the vast majority of cases (80–90%), and no clinical interpretation of TDM results was provided [17]. Lack of bioanalytical experts and that of clinical pharmacological experts coupled with difficult in providing a timely TAT were identified as major barriers for implementing a routinely dedicated CPU for antimicrobial dosing optimization. This is especially true when dealing with septic critically ill patients, for whom short TATs and proper interpretation of TDM results are crucial for timely tailoring antimicrobial therapy [17–19].

In this regard, all of the indicators of performance analyzed in the assessment study period support the positive impact and the clinical usefulness that the ECPA program had in clinical practice.

Notably, the more than 13-fold increase in antimicrobial TDM applications observed after the establishment of the ECPA program highlights the remarkable clinical usefulness that expert interpretation of TDM results had in the critical care setting.

The finding that the ECPAs recommended dosing adjustments in almost two-thirds of first TDM assessments may support the remarkable role that this novel approach may have in allowing early optimization of antimicrobial exposure. This was especially relevant for those beta-lactams that are extensively used in the ICU setting, namely piperacillin–tazobactam and meropenem. It should be noticed that most of the ECPA adaptations recommended dosing decreases. This may be explained by the fact that at our institution beta-lactams are usually administered by continuous infusion in the critically ill patients. Continuous infusion may allow the attainment of a specific PK/PD threshold of beta-lactams in terms of time above the MIC with lower doses compared to intermittent dosing administration [4, 34]. Additionally, it may prevent wide fluctuations of serum concentrations, which may lead to high peaks associated with the potential occurrence of neurotoxicity [4, 34]. An additional explanation for the need of dosing decrease at first TDM assessment is that the dosing schedules of beta-lactams implemented at our institution in critically ill patients are usually very aggressive (i.e., 1 g q6h over 6 h for meropenem). The intent is that of overcoming the major pathophysiological/iatrogenic factors that may cause potential underexposure in the early phase of septic shock (i.e., extensive fluid resuscitation, hyperdynamic status, and transient AKI, application of CRRT) [11, 35].

The finding of an overall need of antimicrobial dosing adjustments in almost half of cases may support the relevant role that the ECPA program had in optimizing antimicrobial exposure during the whole treatment period. Pathophysiological alterations of critically septic patients commonly lead to changes in volume of distribution and clearance of hydrophilic antimicrobials with sudden variations in both plasma and tissue concentrations during the entire treatment course [4, 5, 9]. Fluctuations in renal function during ICU stay may range from ARC to severe AKI with the need of CRRT or vice versa in a given septic patient, and this may clearly affect the likelihood of attaining optimal PK/PD targets. This is especially true for beta-lactams [6], which accounted for almost 70% of delivered ECPAs. In this scenario, close reassessments of antimicrobial concentrations over time become crucial and may result in a non-negligible proportion of dosing adjustments, as reported in our analysis.

It is worth noting that in most cases the TAT of the TDM-guided ECPAs was optimal, namely < 12 h. This allowed intensive care physicians to promptly adapt dosing in different challenging scenarios. On the one hand, this approach may minimize the risk of antimicrobial underexposure potentially associated with clinical failure and/or resistance development [12, 36, 37]. On the other hand, it may prevent the occurrence of overexposure potentially associated with toxicity, which may be especially relevant for narrow therapeutic index antimicrobials, like linezolid [12, 36, 37].

Optimal TATs allowed TDM reassessment every 48–72 h in most cases. In this scenario, implementing a coordinated multidisciplinary taskforce becomes essential [24, 38, 39], and the MD Clinical Pharmacologist should necessarily move from the bench to the bedside for establishing a direct and useful on field relationship with the intensive care physicians. This strategy was helpful in allowing timely identification of sudden pathophysiological alterations and/or in updating microbiological culture results. Expert interpretation of TDM reassessments was fundamental in optimizing the therapeutic strategy in each single patient. Unfortunately, the median TATs of ECPAs for fluconazole, voriconazole, and levofloxacin were quasi-optimal (i.e., 12–24 h). This was not due to any specific issue about expert interpretation, but simply to logistic aspects since in some cases blood collection for TDM was drawn in the early afternoon.

We recognize that our study has some limitations. The retrospective nature of the study design and the short duration of the assessment phase must be acknowledged. The relationship between antimicrobial exposure and clinical outcome was not assessed, even if this was out of the aims of our study. The resources provided in this study could seem very generous. No ECPAs were requested for 5 out of the 15 antimicrobials included in the TDM-guided ECPA program. This is in agreement with the fact that all of these antimicrobials are not widely used in the ICU setting. Posaconazole and isavuconazole are commonly used as chronic therapy or long-term prophylaxis of invasive fungal infections in hematological patients [40]. Ampicillin and or ampicillin–sulbactam have a narrower spectrum of activity, not including particularly *Pseudomonas aeruginosa* [41]. Ceftazidime is preferred over cefepime due to a lower risk of neurotoxicity especially in critically ill renal patients [42]. However, it is worth mentioning that the CPU of our University tertiary hospital has been established with the intent of planning a very ambitious and extensive ECPA program in the metropolitan area of Bologna. The starting core was the ICU settings within our hospital as the critically ill patients have the highest need for prompt optimization of antimicrobial treatment. Indeed, nowadays the ECPA program has just been extended hospital wide, and, in the near future, the project is of furtherly extending it to the other three major city hospitals. Finally, we are aware that using precise MIC value for calculating PK/PD target attainment with antimicrobials may sometimes be imprecise due to some variability associated with MIC determination, especially with automated testing methods [43]. Conversely, the large sample size, the relevant number of delivered ECPAs, and the remarkable proportion of recommended dosing adjustments coupled with the optimal TATs of ECPAS strengthen the feasibility and the clinical usefulness of this novel approach.

In conclusion, our study described for the first time the organizational procedures for establishing a TDM-guided ECPA program and assessed the clinical impact of this approach in tailoring antimicrobial therapy in a large cohort of critically ill patients. The findings suggest that multidisciplinary approach and timely expert interpretation of TDM results by well-trained MD Clinical Pharmacologist could represent cornerstones in improving the cost-effectiveness of the program. Further prospective studies investigating the impact of a real-time TDM-based ECPA program on clinical and microbiological outcomes in ICU patients are warranted.

## Data Availability

The datasets generated and/or analyzed during the current study are not publicly available due to privacy concerns but are available from the corresponding author on reasonable request.

## Abbreviations

AKI: Acute kidney injury
ARC: Augmented renal clearance
BMI: Body mass index
CI: Continuous infusion
CLCr: Creatinine clearance
C_max_: Peak concentration
C_min_: Trough concentration
*C*_ss_: Steady-state concentration
CPS: Clinical pharmacology service
CRRT: Continuous renal replacement therapy
ECPA: Expert clinical pharmacological advice
ICU: Intensive care unit
IQR: Interquartile range
LC–MS/MS: Liquid chromatography–tandem mass spectrometry
LUM: Unique Metropolitan Laboratory
MDR: Multidrug resistant
PK/PD: Pharmacokinetic/pharmacodynamic
SOFA: Sequential organ failure assessment
TAT: Turnaround time
TDM: Therapeutic drug monitoring
